# p104 Binds to Rac1 and Reduces Its Activity during Myotube Differentiation of C2C12 Cell

**DOI:** 10.1155/2014/592450

**Published:** 2014-01-23

**Authors:** Ki Young Choi, Min Sup Lee, Young Jun Cho, Myong Ho Jeong, Seung Jin Han, Seung Hwan Hong

**Affiliations:** ^1^Institute of Molecular Biology and Genetics, Seoul National University, Seoul 151-742, Republic of Korea; ^2^School of Biological Sciences, Seoul National University, San 56-1 Shillim-dong, Kwanak-gu, Seoul 151-742, Republic of Korea; ^3^School of Biological Sciences, Inje University, Gimhae 621-749, Republic of Korea

## Abstract

The p104 protein inhibits cellular proliferation when overexpressed in NIH3T3 cells and has been shown to associate with p85*α*, Grb2, and PLC*γ*1. In order to isolate other proteins that interact with p104, yeast two-hybrid screening was performed. Rac1 was identified as a binding partner of p104 and the interaction between p104 and Rac1 was confirmed by immunoprecipitation. Using a glutathione S-transferase (GST) pull-down assay with various p104 fragments, the 814–848 amino acid residue at the carboxyl-terminal region of p104 was identified as the key component to interact with Rac1. The CrkII which is involved in the Rac1-mediated cellular response was also found to interact with p104 protein. NIH3T3 cells which overexpressed p104 showed a decrease of Rac1 activity. However, neither the proline-rich domain mutant, which is unable to interact with CrkII, nor the carboxy-terminal deletion mutant could attenuate Rac1 activity. During the differentiation of myoblasts, the amount of p104 protein as well as transcript level was increased. The overexpression of p104 enhanced myotube differentiation, whereas siRNA of p104 reversed this process. In this process, more Rac1 and CrkII were bound to increased p104. Based on these results, we conclude that p104 is involved in muscle cell differentiation by modulating the Rac1 activity.

## 1. Introduction

The p104, a cell proliferation regulator, was initially identified as a binding protein of phospholipase C*γ*1 (PLC*γ*1). It can also interact with the p85*α* subunit of phosphoinositide 3-kinase (PI3K) via its proline-rich (PXXP) motifs [1]. Expression of p104 is increased upon serum starvation, and ectopic overexpression of p104 reduces the growth rate of NIH3T3 cells. This growth inhibition can also be achieved by the overexpression of only the proline-rich region of p104. Interestingly, overexpression of p104 leads to increased levels of p27^kip1^, a cyclin-dependent kinase inhibitor, and it inhibits the activities of PI3K that are required for cellular proliferation [1].

Rho GTPases are members of the Ras superfamily, of which more than eleven different mammalian Rho GTPases have been identified; Rho, Rac, Cdc42, Rnd1/Rho6, Rnd2/Rho7, Rnd3/RhoE, RhoG, RhoBTB, RhoD, TTF/RhoH, and Rif [2]. Like all members of the Ras superfamily, the Rho GTPases cycle between two conformational states; GTP- (“active” state) and GDP-bound forms (“inactive” state), by hydrolyzing GTP to GDP. The transition between these two states is tightly regulated by three distinct families of proteins including guanine nucleotide exchange factors (GEFs), GTPase-activating proteins (GAPs), and the guanine nucleotide dissociation inhibitors (GDIs). GEFs activate Rho proteins by catalyzing the exchange of GDP to GTP, whereas there are over 80 Rho GAPs which negatively regulate GTPase function by increasing the intrinsic GTPase activity of Rho proteins [3, 4]. A family of Rho GDIs also negatively regulates Rho GTPases by binding to the GDP-bound form and maintaining it in this inactive state [5].

Rac1, one of the subfamily member of Rho GTPases, plays a pivotal role in many cellular processes including myotube fusion [6], differentiation [7, 8], actin dynamics [9], superoxide production [10, 11], cell movement [12, 13], proliferation [14–16], apoptosis [17–19], and gene expression [18, 20–23], as well as the induction of membrane ruffling and lamellipodia formation upon growth factor stimulation [24]. Rac1 is also required for Ras-induced transformation [8, 25] and constitutive activation of Rac1 causes anchorage-independent growth, invasion, and metastasis [16, 26, 27]. The various cellular functions of Rac1 are achieved through direct or indirect interactions with multiple effector proteins including p21-activated kinase (PAK), MEKK serine/threonine kinases, PI4P5K, PI3K, PLC*β*2, and p67^phox^ [28]. Rac1 has an amino-terminal effector-binding domain encompassing amino acids 26–45 which is essential for the induction of actin polymerization as well as interaction with target enzymes such as PAK and NADPH oxidase [29]. The carboxy-terminal hypervariable domain of Rac1 at residues 143–175 has been implicated in membrane association and effector binding [30]. More recently, a third effector site was identified at residues 124–135. Referred to as the insert region, it is involved in direct interaction with effectors controlling the actin reorganization and cellular transformation [31].

Rac1 is activated by CrkII through DOCK180, a guanine nucleotide exchange factor [32–34]. Crk was originally isolated as the oncogene fusion product of the CT10 chicken retrovirus [35] and the CrkII protein contains an amino-terminal SH2 domain and two SH3 domains [36]. The amino-terminal SH3 domain of the CrkII interacts with several proteins that contain a PXXPXK binding motif, including C3G (a nucleotide exchange factor for Rap1), DOCK180, as well as the Abl tyrosine kinase, tyrosine phosphatase, the p85 subunit of PI3K, and c-Jun N-terminal kinase (JNK) [37–39]. Despite the lack of an enzymatic kinase domain, CrkII is thought to play a crucial role in growth factor-stimulated signal transduction and regulation of actin cytoskeleton [40, 41].

In this study, Rac1 was identified as a new interacting partner of p104 using a yeast two-hybrid screening system. p104 was also shown to be involved in reducing the activity of Rac1 and implicated in myotube differentiation of C2C12 cells by regulating Rac1 activity.

## 2. Materials and Methods

### 2.1. Cell Culture and Transient Transfection

NIH3T3 murine fibroblast and C2C12 murine myoblast were maintained at 37°C in 5% CO_2_ in Dulbecco's Modified Eagle's Medium (DMEM) supplemented with 10% calf serum and 10% fetal bovine serum, respectively. To generate myotubes, C2C12 cells were cultured in DMEM supplemented with 2% horse serum (differentiation medium) for five days. Transient transfection of cells with mammalian expression vectors or siRNA was performed using the Nucleofector System (Amaxa GmbH, Cologne, Germany) according to the manufacturer's guidelines (Table 1).

### 2.2. Plasmid Construction and Expression of Fusion Proteins

The p104I (amino acid residues 7–352), p104II (353–609), and p104III (611–898) constructs were amplified by PCR, incorporating 5′-*Eco*RI and 3′-*Hin*dIII sites and subsequently subcloned into pCMV Taq2B (Stratagene, La Jolla, CA) and pGEX4T1 vectors (GE Healthcare Bio-Sciences, Piscataway, NJ). The p104III-1 (611–726), p104III-2 (698–814), p104III-3 (783–898), p104III-3A (783–848), and p104III-3B (870–898) were amplified and subcloned into *Kpn*I*/Eco*RI sites of pGEX4T1. The C-terminal truncation mutants of RhoA and Cdc42 were amplified by PCR from mouse RhoA and Cdc42 cDNA as templates and the product was subcloned into HindIII/XhoI sites of pEGFP-C1 (Clontech, Palo Alto, CA). Various GST fusion proteins were induced and purified according to previously described procedures [42].

### 2.3. Yeast Two-Hybrid Screening

Yeast two-hybrid screening was performed using the MATCHMAKER yeast two-hybrid system (Clontech) according to the manufacturer's protocols. Briefly, p104 cDNA (amino acids 140–899) was cloned into pGBT9 to produce a fusion protein containing galactosidase-4 (GAL4) DNA-binding domain. This p104 construct and plasmids encoding the adult mouse brain cDNA library fused to the GAL4 activation domain were then transformed into the yeast strain Y190. Positive colonies which grew on plates lacking His, Leu, and Trp were selected. Interaction was further confirmed by a *β*-galactosidase filter assay. Plasmid DNAs were isolated from positive yeast colonies, transformed into *E. coli* and the nucleotide sequences of the positive clones were determined.

### 2.4. Fluorescence Microscopy

NIH3T3 cells were grown on coverslips in 6 well plates and transfected using FuGENE 6 (Roche Diagnostics, Indianapolis, IN) with the pEGFP C1-p104 plasmid. After 24 h, cells were washed twice with PBS and then fixed with 4% paraformaldehyde or methanol (in 1x PBS) for 30 min followed by permeabilization with 0.3% Triton X-100 (in 1x PBS) for 20 min. The coverslips were blocked with 0.5% bovine serum albumin and incubated with Rac1 or lamin A/C antibody (Santa Cruz Biotechnology, Santa Cruz, CA) for 2 h at 4°C followed by Rhodmaine-conjugated anti-rabbit antibody (Jackson ImmunoResearch, West Grove, PA).

### 2.5. Antibodies and Western Blot Analysis

A rabbit antibody to p104 was generated against a p104 fragment (residue 450–803) as described previously [1]. Antibodies for FLAG (M2) and GFP (clones 7.1 and 13.1) were purchased from Sigma-Aldrich Co. (St. Louis, MO) and Roche Diagnostics, respectively. Antibodies for CrkII (sc289) were obtained from Santa Cruz Biotechnology (Santa Cruz, CA). Anti-mouse or anti-rabbit IgG conjugated to horseradish peroxidase was purchased from Jackson ImmunoResearch (West Grove, PA). The expression of the constructs was determined by immunoblotting according to previously published methods [1].

### 2.6. Immunoprecipitation and GST Pull-Down Assay

Cultured cells were collected and washed with PBS and suspended in extraction buffer (25 mM HEPES (pH 7.4), 50 mM NaCl, 1% Triton X-100, 10% glycerol, 0.1 mM PMSF, 0.1 mM pepstatin, 0.1 mM antipain, 0.2 mM leupeptin, 10 *μ*g/mL aprotinin, 1 mM benzamidine, 15 mM NaF, and 1 mM Na_3_VO_4_). To prepare the mouse brain extract, tissue was dissected and grinded using a Polytron homogenizer. In both cases, the extracts were then incubated on ice for 15 min. After lysates were centrifuged at 14,000 ×g for 10 min, clarified proteins (2 mg) were incubated with the appropriate antibodies for 3 h at 4°C with continuous agitation. To collect immune complexes, 30 *μ*L of protein A Sepharose 4B (GE Healthcare Bio-Sciences) was added to the mixture and incubated for a further 2 h. For the GST pull-down assay, glutathione-Sepharose 4B (GE Healthcare Bio-Science) beads bound with GST fusion proteins were incubated with prepared cell or brain extract. The immunoprecipitates or GST complexes were washed three times with extraction buffer and then resuspended in 1x SDS-PAGE sample buffer. Proteins bands were detected by immunoblotting.

### 2.7. RT-PCR

Total RNA was isolated from C2C12 cells using TRIzol (Invitrogen, Carlsbad, CA). Two micrograms of total RNA was primed with a mixture of 0.5 *μ*g oligo-dT primer and mixed with 10 mM DTT, 10 mM of each dNTP, 5x first strand buffer, and MMLV reverse transcriptase (Promega, Madison, WI) for reverse transcription. Using these mixtures as templates, PCR was performed with primer sets listed below; Primers for p104 were sense, 5′ GGT GAA GAA GCT GAA GGA G 3′; antisense, 5′ GGA TTT TCA GGG ATT TCT AC 3′. Primers for Myogenin were sense, 5′ ATG GAG CTG TAT GAG ACA 3′; antisense, 5′ CTC CTG GGT TGG GAC CGA 3′. Primers for *α*-Actin were sense, 5′ CAC CAA GGT GTC ATG GTA 3′; antisense, 5′ GCT CTT CTC CAA GGA GGA 3′.

### 2.8. Rac1 Activity Assay

For affinity precipitation of GTP-bound Rac1, cells were washed with ice-cold PBS and incubated with the extraction buffer. The cleared lysates were then incubated with GST-PAK (25 *μ*g) precoupled to glutathione-Sepharose for 1 h with continuous agitation. The beads were washed three times with wash buffer (20 mM HEPES [pH 7.5], 10 mM potassium acetate, 50 mM NaCl, 1 mM EGTA, 10% glycerol, and 50 mM NaF). Beads were resuspended in reducing 1x SDS sample buffer and boiled for 3 min. Samples were run on 12% SDS-PAGE gels before immunodetection was performed with an anti-Rac1 antibody.

### 2.9. JNK Activity Assay

Using Radioimmunoprecipitation (RIPA) buffer (25 mM Tris HCl, pH 7.6, 150 mM NaCl, 1% NP-40, 1% sodium deoxycholate, 0.1% SDS), cell extracts were incubated for 2 h at 4°C with 0.5 *μ*g of anti-JNK1 antibody (Santa Cruz Biotechnology). The JNK1 antibody was recovered by the addition of 30 *μ*L of protein A-Sepharose beads (0.03 g/mL) and incubated for 2 h at 4°C. Beads were collected by centrifugation and washed three times with RIPA buffer. Kinase assays were initiated by the addition of reaction buffer (2 mM Na_3_VO_4_, 20 mM DTT, and 100 mM MgCl_2_), 3 *μ*g GST c-Jun, and [*γ*-^32^P] ATP (6,000 Ci/mmol). After incubation for 1 hour at 30°C, reactions were terminated by the addition of an equal volume of 2x SDS sample buffer and boiling. Phosphorylated c-Jun was resolved by 12% SDS-PAGE gels and visualized by autoradiography. To check the effect of p104 overexpression on ERK activity, serum-starved cells were treated with 50 ng/mL PDGF BB for 5 min. Fifty mg of each cell lysate was separated on 10% SDS-PAGE and analyzed by immunoblot analysis using a phosphorylated form specific Erk antibody (Cell Signaling Tech. Inc., Danvers, MA).

## 3. Results

### 3.1. Identification of Rac1 as a Binding Partner of p104

**Figure 1:**
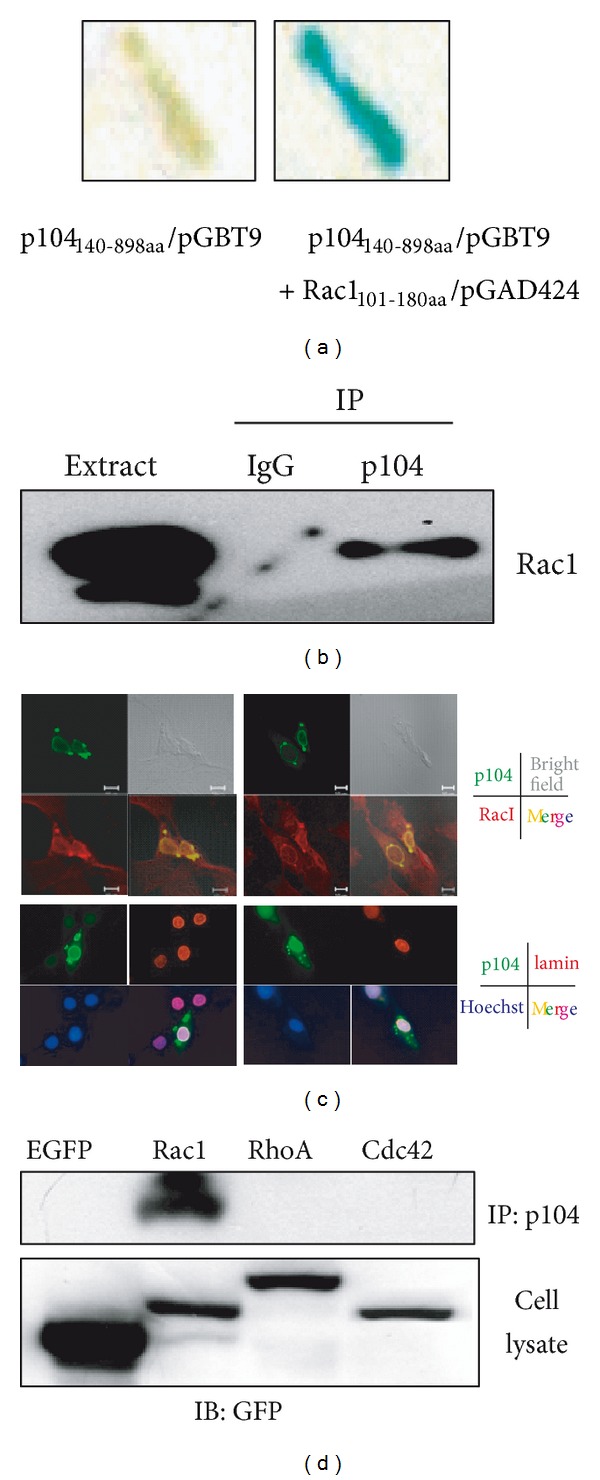
Rac1 was isolated as a binding protein of p104. (a) The DNA-encoding p104 (amino acids residue 140–898) was inserted into the *Eco*RI/*Pst *Isite of a GAL4 DNA-binding domain vector pGBT9. After colony selection on plates lacking Trp, His, and Leu, *β*-galactosidase assay was performed on the filter. Rac1 clones were isolated as a binding partner of p104. (b) Using anti-p104 serum, immunoprecipitation was performed with mouse brain extract and analyzed by Western blotting with an anti-Rac1 antibody. Immunoprecipitation using an unrelated antibody (IgG) was used as a negative control. (c) Colocalization of p104 and Rac1 in NIH3T3 cells. NIH3T3 cells were grown on cover slips in 6 well plates and transfected with the pEGFP C1-p104 plasmid. After 24 h, cells were fixed and stained with a Rac1 or lamin antibody followed by a Rhodamine-conjugated anti-rabbit antibody. Fluorescence was observed using a confocal microscope (upper eight panels) or fluorescence microscope (lower eight panels). (d) p104 was specifically associated with Rac1. The carboxy-terminal domain of Rac1, RhoA and Cdc42 was fused with EGFP and transfected into C2C12 cells. After 24 h, cell lysates were immunoprecipitated with anti-p104 serum and Western blot analysis was performed using an anti-GFP antibody. The expression of EGFP-fused Rac1, RhoA, and Cdc42 is shown as a control in the lower panel.

To isolate the protein(s) that interact with p104, yeast two-hybrid screening was employed with p104 (amino acids 140–899) as a bait in combination with the mouse brain cDNA library. The bait contains proline-rich region of p104, which interacts with PLC*γ*1 and p85*α*, as well as spectrin repeats [1]. From over 40,000 colonies screened, 9 positive clones that specifically interacted with p104 were isolated and their nucleotide sequences were determined. One of the isolated clones was found to be Rac1 (Figure 1(a)), which is widely known to play a key regulatory role in various cellular processes such as proliferation, apoptosis, and gene expression as well as cell movement [10, 12–17, 19–21]. When immunoprecipitation analysis was performed with mouse brain extracts, Rac1 was detected in the coimmunoprecipitated fraction with an antibody against p104 (Figure 1(b)).

The localization of p104 and Rac1 in intact cells was investigated by fluorescent and confocal microscopy. The EGFP-p104 was predominantly localized to the nuclear membrane where lamin protein is present (Figure 1(c), lower panel). The staining pattern for p104 and Rac1 confirmed *in vivo* colocalization of these two proteins in NIH3T3 cells (Figure 1(c), upper panel). p104 was also found in the cytoplasm of transfected cells as a strong fluorescent structure which remains to be identified.

The peptide region in Rac1 that was isolated as a binding partner of p104 by yeast two-hybrid screening (residues 101–180) contains a polybasic region which is common to the carboxy-terminus of Ras and Rho family members. The region promotes interaction with other binding partners [43]. To determine whether p104 could also interact with the polybasic region of other Rho GTPases, GFP-fused carboxy-terminal fragments of Rac1, RhoA, and Cdc42 were transfected into NIH3T3 cells. Only Rac1 and not RhoA or Cdc42 coimmunoprecipitated with p104 (Figure 1(d)), indicating that p104 interacts specifically with Rac1.

### 3.2. The Carboxy-Terminal Region of p104 Is Involved in the Interaction with Rac1

**Figure 2:**
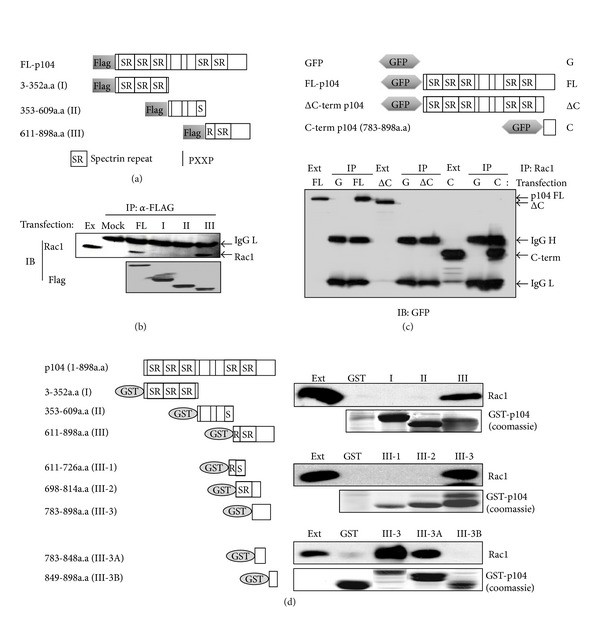
The carboxy-terminal region of p104 was essential for the interaction with Rac1. (a) Schematic representation of the constructs used in this experiment. p104 was divided into three regions and inserted into the pCMV Taq2B vector. Full-length (FL) 104 contains the entire open reading frame (ORF), and constructs I, II, and III contain 7–352, 353–609, and 611–898 amino acid residues, respectively. (b) p104 interacts with Rac1 through the carboxy-terminal 611–898 amino acid region. NIH3T3 cells were transfected with a Flag-tagged p104 construct. After 24 h, 2 mg of clarified cell lysates was immunoprecipitated with an anti-Flag antibody followed by Western blot analysis using an anti-Rac1 antibody (upper panel). The immunoprecipitated Flag-tagged protein was confirmed with an anti-FLAG antibody (lower panel). (c) The carboxy-terminal region of p104 is required for the association with Rac1 in mammalian cells. Full-length p104 (FL), carboxy-terminal deleted p104 (ΔC), and carboxy-terminus of p104 (C) were inserted into a GFP expression vector (G), and the constructs were transfected into NIH3T3 cells. Twenty-four hours after transfection, the interaction between various truncated forms of p104 and Rac1 was assayed by immunoprecipitation with the anti-Rac1 antibody followed by Western blot analysis using an anti-GFP mouse monoclonal antibody. Ext: cell extract, IgG H: immunoglobulin heavy chain, and IgG L: immunoglobulin light chain. (d) The 814–848 residue of p104 is required for the association with Rac1. Two micrograms of GST-fused p104 I, II, and III was used for the GST pull-down assay. Prepared GST-fused p104 I, II, and III were incubated with 1 mg of mouse brain extract, and bound proteins were then subjected to Western blot analysis to detect Rac1. p104 III (amino acid residues 611–898) was subdivided into three parts, consisting of residues 611–726 (III-1), 698–814 (III-2), and 783–898 (III-3). Each DNA fragment was inserted into a pGEX 4T1 vector and a GST-pull-down assay was carried out. Again, p104III-3 was divided into III-3A (783–848) and III-3B (870–898) and then inserted into the pGEX 4T1 vector. Each construct was used in the *in vitro* binding assay as described above. A coomassie brilliant blue stained SDS-PAGE gel showed that equal amounts of fusion proteins were used.

The fragments of FLAG-tagged p104 (Figure 2(a), amino acids 3–352, 353–609, and 611–898) were transfected into NIH3T3 cells and immunoprecipitated with an anti-FLAG antibody. As shown in Figure 2(b), Rac1 coimmunoprecipitated with full-length p104 and the fragment containing amino acids 611–898. This result was confirmed by *in vivo* interaction between p104 and Rac1 through the carboxy-terminal region of p104. When a carboxy-terminal deletion mutant or the carboxy-terminal fragment of GFP-fused p104 was transfected into NIH3T3 cells, Rac1 coimmunoprecipitated with the constructs containing the carboxy-terminal region of p104 (Figure 2(c), lanes 3, 9) while the deletion of the carboxy-terminal region abolished the interaction with Rac1 (Figure 2(c), lane 6). To define the region of p104 responsible for interaction with Rac1, various GST-fused p104 constructs were prepared and a GST pull-down assay was performed. Mouse brain extract was incubated with various immobilized GST-fused p104 constructs and bound protein complexes were subjected to immunoblotting with a Rac1 antibody. The GST pull-down assay revealed that the carboxy-terminus of p104, specifically the short fragment containing amino acids 814–848, could interact with Rac1 (Figure 2(d)). These results indicate that this 34 amino acid fragment in the carboxy-terminal region of p104 is necessary for interaction with Rac1 *in vivo*.

### 3.3. p104 Also Interacts with CrkII in addition to Rac1

**Figure 3:**
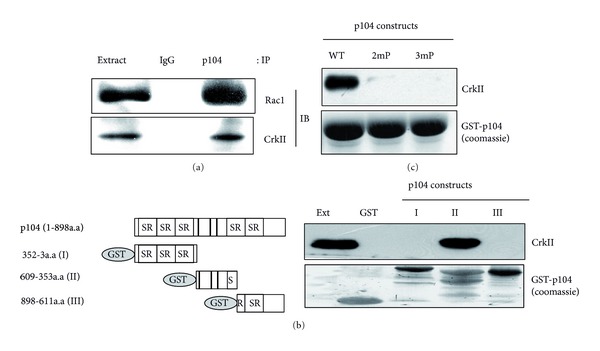
p104 was specifically associated with Rac1. (a) p104 interacted with CrkII as well as Rac1. After immunoprecipitation was performed with the p104 antibody, the presence of CrkII and Rac1 in the precipitated immune complex was analyzed by Western blot with anti-CrkII and anti-Rac1 antibodies, respectively. (b) CrkII and Rac1 bind to distinct regions of p104. GST-fused p104 I, II, and III proteins linked to glutathione-Sepharose beads were incubated with mouse brain extract and then bound proteins were analyzed by Western blot with antibodies against CrkII and Rac1. (c) p104 directly interacts with CrkII through the proline-rich region. Mouse brain extract was incubated with GST-p104 (WT, wild type), GST-2mp (second proline-rich motif mutant), and GST-3mp (third proline-rich motif mutant) immobilized onto glutathione-Sepharose beads. Bound proteins were analyzed by Western blot with an anti-CrkII antibody. A coomassie brilliant blue stained SDS-PAGE gel showed that equal amounts of fusion proteins were used.

The adapter protein CrkII mediates the formation of multiprotein signaling complexes in response to a variety of extracellular stimuli and regulates cytoskeleton dynamics, cell motility, and phagocytosis in a Rac1-dependent manner [34, 44, 45]. To further understand the roles of p104 in the Rac1 signaling pathway, the association of p104 with CrkII was investigated. As shown in Figure 3(a), CrkII could be coimmunoprecipitated with p104 just like Rac1. To define more precisely which region within the p104 protein interacts with CrkII, a GST pull-down assay was carried out with GST-fused p104 fragments. As shown in Figure 3(b), CrkII could bind to the central region of p104 (amino acids 353–609). The central region of p104 contains three proline-rich (PXXP) motifs that are known to interact with the SH3 domain. Since CrkII contains an SH2 and two SH3 domains, it was investigated whether p104 directly interacts with CrkII through its PXXP motifs. As the second and third proline-rich motifs in p104 are essential for the cell cycle inhibitory function of p104 [1], point mutations were introduced in the second or third PXXP motifs (2mP and 3mP) and binding activities with CrkII were tested in the GST pull-down assay. As shown in Figure 3(c), mutations in the PXXP motifs resulted in a dramatic decrease in the binding activity with CrkII, suggesting that the interaction between p104 and CrkII occurs through the PXXP motifs of p104.

### 3.4. p104 Decreases Rac1 Activity

**Figure 4:**
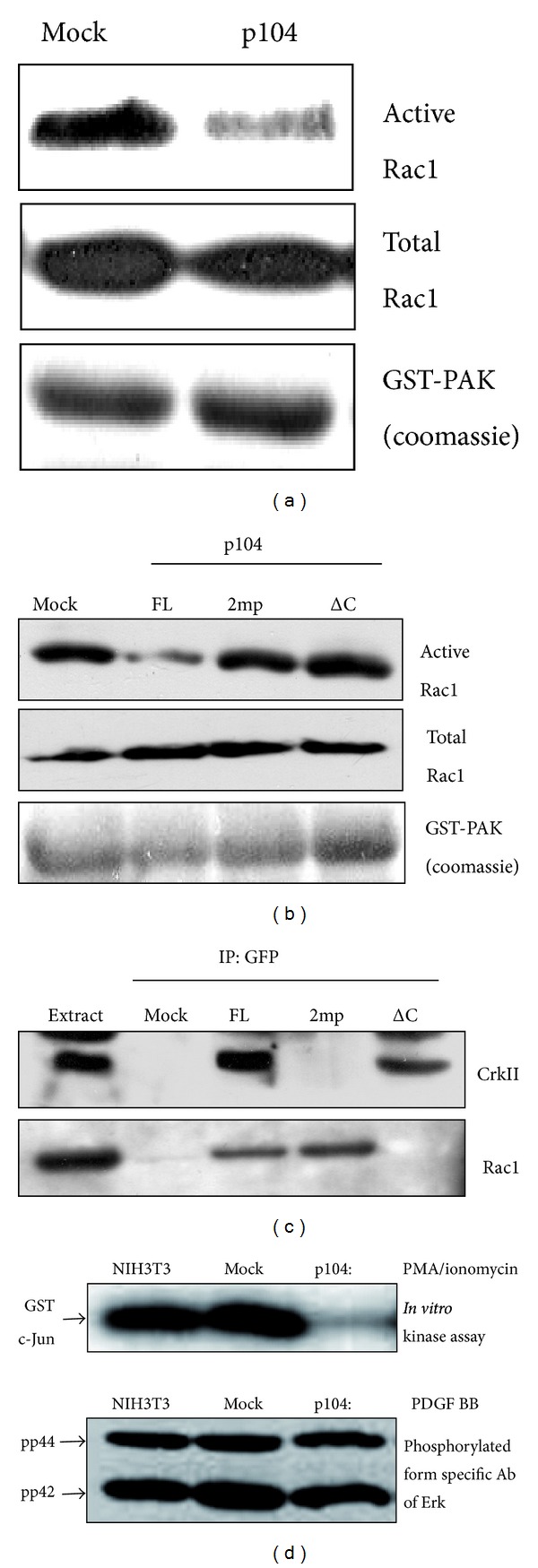
The interaction of p104 with CrkII decreased the activity of Rac1. (a) NIH3T3 cells were transfected with empty vector (Mock) or p104 constructs. After 24 h of transfection, the amount of GTP-bound (active) Rac1 was determined by GST-PAK pull-down analysis, followed by Western blotting with an anti-Rac1 antibody (upper panel). 50 *μ*g of cell lysate was used for Western blot analysis with an anti-Rac1 antibody to check the total amount of Rac1 (middle panel). The amount of GST-PAK used in this assay was assessed by coomassie brilliant blue staining (bottom panel). (b) NIH3T3 cells were transfected with GFP-tagged full-length p104 (FL), second proline-rich motif mutant (2mp) and carboxy-terminal deletion mutant (ΔC). The level of GTP-bound Rac1 was measured by GST-PAK pull-down analysis, followed by Western blotting with anti-Rac1 antibody. (c) Lysates from NIH3T3 cells transfected with GFP-tagged full-length p104, 2mp mutant, and carboxy-terminal deletion mutant were immunoprecipitated with an anti-GFP antibody, and then Western blot analysis was performed with anti-CrkII (upper panel) and anti-Rac1 antibodies (lower panel). (d) Inhibition of JNK activity by p104 overexpression. To measure the JNK activity, cells were serum-starved for 12 h and then treated with 5 *μ*M PMA/ionomycin for 5 min. JNK was immunoprecipitated and kinase assays were performed using GST c-Jun as a substrate (upper panel). After treatment with 50 ng/mL PDGF BB, 50 *μ*g of each cell lysate was separated on SDS-PAGE gel and they were analyzed by Western blot using an anti-active ERK antibody to check the Erk activity (lower panel).

One of the well-known characteristics of Rho GTPases is that they bind to their effectors in an active GTP-bound form. This property was used to measure the relative amount of GTP-bound GTPases in cells by pull-down experiments with the GTPase binding domain of PAK. To investigate the influence of p104 on the activity of Rac1, wild-type p104 was transfected into NIH3T3 cells. When the GTP-bound (active) form of Rac1 was precipitated using GST-PAK fusion proteins precoupled to GSH-beads, the amount of GTP-bound Rac1 was decreased in wild-type p104 transfectant (Figure 4(a)). The mutant in second proline-rich motif (2mP) and ΔC-terminal mutant of p104 could not attenuate the Rac1 activity, unlike the wild-type p104 transfectant (Figure 4(b)). The mutation in the proline-rich motif of p104 did not affect the binding capacity with Rac1, whereas carboxy-terminal deletion of 104 could not interrupt the interaction with CrkII (Figure 4(c)). Taken together, these results suggest that the interaction of p104 with CrkII as well as Rac1 might be a critical determinant for the regulation of Rac1 activity.

Since it has been reported that Rac1 activates the mitogen-activated protein kinase JNK [14, 19, 46] and phorbol 12-myristate 13-acetate (PMA), a well-known stimulator for JNK induces the phosphorylation of p104 [1]; the relationship between p104 and Rac1 in JNK activation was subsequently analyzed. When cells were treated with PMA, the degree of phosphorylation of GST-Jun, a substrate of active JNK, was reduced in the p104 overexpressed NIH 3T3 cells (Figure 4(d), upper panel). However, the activation of Erk was not affected by p104 overexpression upon the addition of PDGF BB, an Erk signal cascade stimulator (Figure 4(d), lower panel). Thus, our results suggest that p104 may inhibit JNK activity through the reduction of Rac1 activity.

### 3.5. More Rac1 Interacts with p104 during Myoblast Differentiation

**Figure 5:**
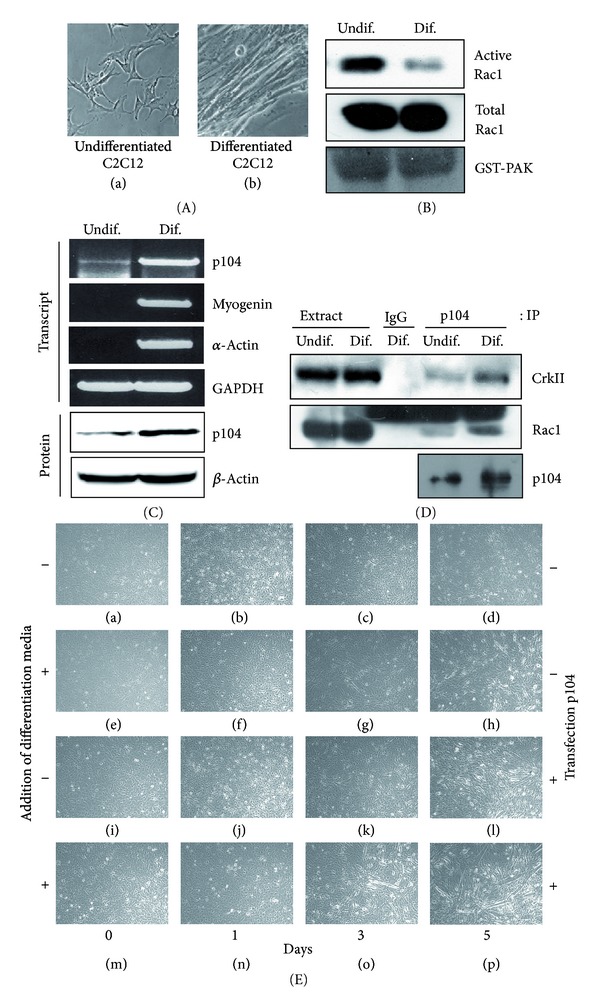
Interaction of p104 with Rac1 and CrkII during C2C12 differentiation. (A) C2C12 cells were grown to confluence (a) and then placed in differentiation medium for 5 days to induce the differentiation into myotube formation (b). (B) The activity of Rac1 was decreased during C2C12 differentiation. The amount of GTP-bound Rac1 in cell lysates prepared from undifferentiated (Undif.) or differentiated (Dif.) myoblasts was analyzed by GST-PAK pull-down analysis. Rac1-GTP (active form) and total Rac1 from the same extracts were detected by Western blotting with Rac1 antibody. (C) Transcript levels of p104, myogenin, and *α*-actin and the protein level of p104 were determined in the absence (Undif.) or presence (Dif.) of differentiation medium by reverse transcription-polymerase chain reaction and Western blot analysis, respectively. The mRNA levels of p104 and differentiation markers were increased 5 days after the induction of differentiation. Levels of glyceraldehydes-3-phosphate dehydrogenase (GAPDH) transcript and actin were used as a control. (D) Interaction of p104 with Rac1 and CrkII was increased during the C2C12 differentiation. C2C12 cells were grown in differentiation media for 5 days and total protein was extracted. Lysates from undifferentiated or differentiated cells were immunoprecipitated with p104 antiserum, and then Western blot analysis was performed using anti-CrkII, anti-Rac1, and anti-p104 antibodies. (E) The effect of p104 overexpression in the myotube differentiation. The C2C12 cells were transfected with p104 ((i)–(p)) and grown in the presence ((e)–(h), (m)–(p)) or absence ((a)–(d), (i)–(l)) of differentiation media. The photos were taken at the indicated days after the addition of differentiation media.

Recently, it was reported that Rho family proteins play a critical role in muscle differentiation. In the absence of mitogenic stimuli, proliferating myoblasts synchronously withdraw from the cell cycle, elongate, adhere, and finally fuse together to form myotubes. The amount of GTP-bound Rac1, the active form, was high in proliferating myoblasts and was significantly reduced after the induction of differentiation [47]. To test the possibility that p104 might be involved in the process of myoblast differentiation, C2C12 myoblast cell differentiation was induced by replacing the growth medium with differentiation medium when cells reached confluence. C2C12 cells underwent morphological change and elongation and formed small myofibers after the addition of differentiation medium (Figure 5(A)). When the Rac1 activity assay was performed, a decrease in Rac1 activity was readily detected after the induction of differentiation (Figure 5(B)). As shown in Figure 5(C), the expression levels of p104 mRNA and protein were elevated after incubation in differentiation medium. The increase in myogenin (a muscle regulatory factor) and *α*-actin (a skeletal muscle-specific gene) transcripts also indicated muscle differentiation. When C2C12 differentiation was induced, more Rac1 and CrkII were shown to interact with p104 (Figure 5(D)), although protein levels of Rac1 and CrkII were unchanged during the differentiation process. These results suggest that p104 might be involved in muscle differentiation by sequestering Rac1 and CrkII and thereby decreasing the active Rac1.

**Figure 6:**
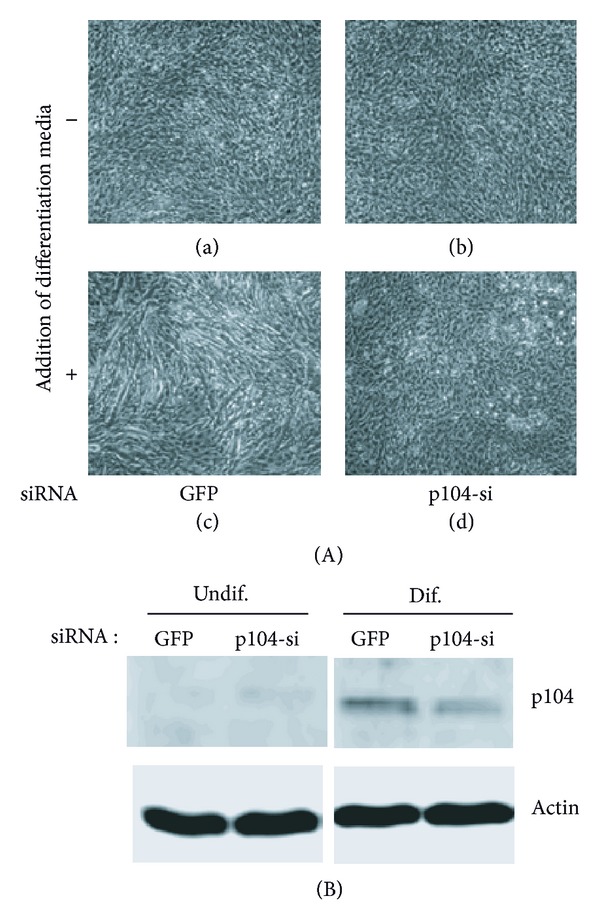
The effect of p104 siRNAs transfection in the myotube differentiation. (A) The C2C12 cells were transfected with p104 siRNA ((b), (d)) and grown in the presence ((c), (d)) or absence ((a), (b)) of differentiation media. The photos were taken 4 days after siRNAs transfection. (B) The decrease of p104 protein in the differentiation condition was shown by Western blot analysis.

To confirm the role of p104 in the myotube differentiation, p104 was overexpressed in the C2C12 cell. In the differentiation media (2% horse serum), the overexpression of p104 caused the induction of differentiation in a shorter period of time compared to nontransfected cells (Figure 5(E), compare (g) with (o)). Interestingly, the degree of differentiation of p104 transfected cells in the nondifferentiation media (10% FBS) is higher than that of nontransfected and differentiation media added cells (Figure 5(E), (h) versus (l)). In contrast, three sets of siRNAs against p104 partially inhibited the myotube formation (Figures 6(A) and 6(B)). This result strongly supports our claim that p104 regulates the myotube differentiation.

## 4. Discussion

In this study, a novel interaction between carboxy-terminal portion of p104 and Rac1 was examined using several methods such as yeast two-hybrid screening, coimmunoprecipitation, and pull-down analysis (Figures 1 and 2). Also, colocalization of p104 and Rac1 proteins in NIH3T3 cells was confirmed by immunocytochemical studies (Figure 1(c)). Given the previous studies about p104 induced growth suppression through the upregulation of p27^Kip1^ in NIH3T3 cells [1] and involvement of Rac1 in G1/S cell cycle transition by inducing cyclin D1 accumulation [48, 49], we could infer the significance of the interaction between the two proteins. Data from this study suggests that Rac1 might be a mediator of the relationship between p104 and p27^kip1^. p27^kip^ inhibits the cyclin D-Cdk4/6 complexes, a key player in G1/S transition [50, 51]. It has been reported that the Rac1 signaling pathway induced by integrin signaling resulted in a rapid reduction of the Cdk inhibitors, p21^CIP1^ and p27^Kip1^, by proteasomal degradation [52]. There is a possibility that p104 may suppress the cell cycle progression by inactivating the Rac1, followed by the downregulation of cyclin D synthesis and inhibition of p27^Kip1^ degradation. Another interesting question to be investigated is whether the interaction between p104 and Rac1 is dependent on the nucleotide binding status of Rac1 (i.e., GTP, GDP, or nucleotide free), since Rac1 is a Rho family small GTPase.

The activity of Rac1 was reduced by overexpression of p104 (Figure 4), even though the molecular mechanism of the reduction of the Rac1 activity is still unclear. The possible explanations are that p104 could be acting as an atypical GAP for Rac1 or that p104 binding to Rac1 prevents GEF access to Rac1. Another explanation is based on our previous studies; overexpression of p104 inhibits the activity of PI3K by interaction with p85*α* through the second and third proline-rich region of p104 [1]. Furthermore, it is known that Rac1 and its effector proteins such as Vav and Sos guanine-nucleotide exchange factors work at the downstream in the growth factor-mediated PI3K pathway [53–56]. Therefore, it is plausible to think that p104 interacts with p85 through its proline-rich domain to inhibit PI3K activity and to subsequently reduce the Rac1 activity.

One of the most interesting findings in this study was that p104 can also interact with CrkII (Figure 3). CrkII is an adaptor protein known to interact with several proteins through either its SH2 or SH3 domains [57]. In this study, it was verified that CrkII can interact directly with p104 through its proline-rich region (Figure 3(c)). Ablation of this site and the carboxy-terminal of p104 functionally impaired the ability of p104 to reduce the activity of Rac1 (Figures 4(a) and 4(b)). These results indicate that these regions are functionally linked with CrkII and Rac1 in reducing the activity of Rac1. Several reports have stated that CrkII preferentially activates the small GTPase Rac1 through DOCK180, a guanine-nucleotide exchange factor, and this regulation is achieved by the formation of a multiple protein complex with docking protein p130Cas [32, 33]. We hypothesize that p104 also functions as a docking protein that can regulate the activity of Rac1 by making a complex with Rac1, CrkII, as well as PI3K. However, this docking protein appears to have a negative role in regulating the activity of Rac1, unlike the p130Cas. Even though it is not clear whether p104 could bind to Rac1 and CrkII simultaneously, it is possible that CrkII and Rac1 are sequestered spatiotemporally by binding to p104 under certain physiological conditions such as serum starvation. Further studies should be carried out to clear this aspect.

The overexpression of p104 facilitated C2C12 differentiation (Figure 5(E)). Myoblasts differentiate into skeletal muscle cells by withdrawal from the cell cycle and subsequent expression of myotube-specific genes [58]. p104 might enhance this differentiation via its ability to block cell cycle because p104 appears to play a key role in cell cycle regulation [1]. In this case, p104 depletion may delay the ability of C2C12 to stop proliferation when the cell was switched to a differentiation medium. Another possibility is related with the reports in which activated Rac1 prevents the withdrawal from the cell cycle and muscle differentiation, while dominant negative form of Rac1 induces differentiation [47]. The inhibitory effect of the constitutively active Rac1 protein on myogenesis is directly linked to the activation of the JNK pathway [59]. The direct interaction of CrkII with JNK is critical for Rac1-induced JNK activation [39]. In this study, expression level of p104 was increased and the activity of Rac1 was decreased after the addition of differentiation medium (Figure 5). Because p104 could interact with CrkII and Rac1 and its amount was increased during the muscle cell differentiation, more CrkII and Rac1 could interact with increased p104 when C2C12 myoblast differentiation was induced (Figure 5(D)). It is possible that an increase in the amount of p104 resulted in sequestering more Rac1 and CrkII from JNK during muscle differentiation. In fact, as shown in Figure 4(d), JNK activity was decreased by overexpression of p104. These data collectively support the idea that p104 may prevent the JNK activation by sequestering and reducing Rac1 activity during muscle differentiation.

## Figures and Tables

**Table 1:** Sequences of siRNA used in p104 knockdown.

GFP	Sense	GUUCAGCGUGUCCGGCGAGTT
	Antisense	CUCGCCGGACACGCUGAACTT

p104-a	Sense	GUGAAGAAGCUGAAGGAGATT
	Antisense	UCUCCUUCAGCUUCUUCACTT

p104-b	Sense	UGGCGUAAGAGGAGAGAGATT
	Antisense	UCUCUCUCCUCUUACGCCATT

p104-c	Sense	GUGAAGAGACUGCAAGAGATT
	Antisense	UCUCUUGCAGUCUCUUCACTT

## Abbreviations

GAP: GTPase-activating protein
GDI: Guanine-nucleotide dissociation inhibitor
GEF: Guanine-nucleotide exchanging factor
GST: Glutathione S-transferase
JNK: c-Jun N-terminal kinase
PAK: p21-activated kinase
PI3K: Phosphoinositide 3-kinase
PLC*γ*1: Phosphoinositol-specific phospholipase C*γ*1
SH2: Src Homology domain 2
SH3: Src Homology domain 3.

## References

[1] Han SJ, Lee JH, Choi KY, Hong SH (2010). Novel p104 protein regulates cell proliferation through PI3K inhibition and p27Kip1 expression. *BMB Reports*.

[2] Heasman SJ, Ridley AJ (2008). Mammalian Rho GTPases: new insights into their functions from in vivo studies. *Nature Reviews Molecular Cell Biology*.

[3] Hall A (1998). Rho GTpases and the actin cytoskeleton. *Science*.

[4] Hall A (1990). The cellular functions of small GTP-binding proteins. *Science*.

[5] Fukumoto Y, Kaibuchi K, Hori Y (1990). Molecular cloning and characterization of a novel type of regulatory protein (GDI) for the rho proteins, ras p21-like small GTP-binding proteins. *Oncogene*.

[6] Vasyutina E, Martarelli B, Brakebusch C, Wende H, Birchmeier C (2009). The small G-proteins Rac1 and Cdc42 are essential for myoblast fusion in the mouse. *Proceedings of the National Academy of Sciences of the United States of America*.

[7] Behrendt K, Klatte J, Pofahl R (2012). A function for Rac1 in the terminal differentiation and pigmentation of hair. *Development*.

[8] Kucharczak J, Charrasse S, Comunale F (2008). R-cadherin expression inhibits myogenesis and induces myoblast transformation via Rac1 GTPase. *Cancer Research*.

[9] Starr DA, Han M (2003). ANChors away: an actin based mechanism of nuclear positioning. *Journal of Cell Science*.

[10] Dorseuil O, Vazquez A, Lang P, Bertoglio J, Gacon G, Leca G (1992). Inhibition of superoxide production in B lymphocytes by Rac antisense oligonucleotides. *Journal of Biological Chemistry*.

[11] Daugaard M, Nitsch R, Razaghi B (20132013). Hace1 controls ROS generation of vertebrate Rac1-dependent NADPH oxidase complexes. *Nature Communications*.

[12] Takaishi K, Kikuchi A, Kuroda S, Kotani K, Sasaki T, Takai Y (1993). Involvement of rho p21 and its inhibitory GDP/GTP exchange protein (rho GDI) in cell motility. *Molecular and Cellular Biology*.

[13] Woo S, Housley MP, Weiner OD, Stainier DY (2012). Nodal signaling regulates endodermal cell motility and actin dynamics via Rac1 and Prex1. *Journal of Cell Biology*.

[14] Minden A, Lin A, Claret F-X, Abo A, Karin M (1995). Selective activation of the JNK signaling cascade and c-Jun transcriptional activity by the small GTPses Rac and Cdc42Hs. *Cell*.

[15] Ma J, Xue Y, Liu W (2013). Role of activated rac1/cdc42 in mediating endothelial cell proliferation and tumor angiogenesis in breast cancer. *PLoS One*.

[16] Guo X, Wang M, Jiang J (2013). Balanced Tiam1-rac1 and RhoA drives proliferation and invasion of pancreatic cancer cells. *Molecular Cancer Research*.

[17] Brenner B, Koppenhoefer U, Weinstock C, Linderkamp O, Lang F, Gulbins E (1997). Fas- or ceramide-induced apoptosis is mediated by a Rac1-regulated activation of Jun N-terminal kinase/p38 kinases and GADD153. *Journal of Biological Chemistry*.

[18] Han P, Luan Y, Liu Y (2013). Small interfering RNA targeting Rac1 sensitizes colon cancer to dihydroartemisinin-induced cell cycle arrest and inhibited cell migration by suppressing NFkappaB activity. *Molecular and Cellular Biochemistry*.

[19] Valente AJ, Yoshida T, Clark RA, Delafontaine P, Siebenlist U, Chandrasekar B (2013). Advanced oxidation protein products induce cardiomyocyte death via Nox2/Rac1/superoxide-dependent TRAF3IP2/JNK signaling. *Free Radical Biology and Medicine*.

[20] Xie W, Herschman HR (1996). Transcriptional regulation of prostaglandin synthase 2 gene expression by platelet-derived growth factor and serum. *Journal of Biological Chemistry*.

[21] Clarke N, Arenzana N, Hai T, Minden A, Prywes R (1998). Epidermal growth factor induction of the c-jun promoter by a rac pathway. *Molecular and Cellular Biology*.

[22] Hill CS, Wynne J, Treisman R (1995). The Rho family GTPases RhoA, Rac1, and CDC42Hs regulate transcriptional activation by SRF. *Cell*.

[23] Montaner S, Perona R, Saniger L, Lacal JC (1998). Multiple signalling pathways lead to the activation of the nuclear factor *κ*B by the Rho family of GTPases. *Journal of Biological Chemistry*.

[24] Gauthier-Rouvière C, Vignal E, Mériane M, Roux P, Montcourier P, Fort P (1998). RhoG GTPase controls a pathway that independently activates Rac1 and Cdc42Hs. *Molecular Biology of the Cell*.

[25] Qiu R-G, Chen J, Kirn D, McCormick F, Symons M (1995). An essential role for Rac in Ras transformation. *Nature*.

[26] Keely PJ, Westwick JK, Whitehead IP, Der CJ, Parise LV (1997). Cdc42 and Rac1 induce integrin-mediated cell motility and invasiveness through PI(3)K. *Nature*.

[27] Lewis-Saravalli S, Campbell S, Claing A (2013). ARF1 controls Rac1 signaling to regulate migration of MDA-MB-231 invasive breast cancer cells. *Cell Signalling*.

[28] Bishop AL, Hall A (2000). Rho GTPases and their effector proteins. *Biochemical Journal*.

[29] Diekmann D, Abo A, Johnston C, Segal AW, Hall A (1994). Interaction of Rac with p67(phox) and regulation of phagocytic NADPH oxidase activity. *Science*.

[30] van Hennik PB, Ten Klooster JP, Halstead JR (2003). The C-terminal domain of Rac1 contains two motifs that control targeting and signaling specificity. *Journal of Biological Chemistry*.

[31] Karnoub AE, Der CJ, Campbell SL (2001). The insert region of Rac1 is essential for membrane ruffling but not cellular transformation. *Molecular and Cellular Biology*.

[32] Kiyokawa E, Hashimoto Y, Kobayashi S, Sugimura H, Kurata T, Matsuda M (1998). Activation of Rac1 by a Crk SH3-binding protein, DOCK180. *Genes and Development*.

[33] Smith HW, Marra P, Marshall CJ (2008). uPAR promotes formation of the p130Cas-Crk complex to activate Rac through DOCK180. *Journal of Cell Biology*.

[34] Feng H, Hu B, Liu K-W (2011). Activation of Rac1 by Src-dependent phosphorylation of Dock180Y1811 mediates PDGFR*α*-stimulated glioma tumorigenesis in mice and humans. *Journal of Clinical Investigation*.

[35] Mayer BJ, Hamaguchi M, Hanafusa H (1988). A novel viral oncogene with structural similarity to phospholipase C. *Nature*.

[36] Matsuda M, Reichman CT, Hanafusa H (1992). Biological and biochemical activity of v-Crk chimeras containing the SH2/SH3 regions of phosphatidylinositol-specific phospholipase C-*γ* and Src. *Journal of Virology*.

[37] Feller SM (2001). CrK family adaptors-signalling complex formation and biological roles. *Oncogene*.

[38] Gelkop S, Babichev Y, Isakov N (2001). T cell activation induces direct binding of the Crk adapter protein to the regulatory subunit of phosphatidylinositol 3-kinase (p85) via a complex mechanism involving the Cbl protein. *Journal of Biological Chemistry*.

[39] Girardin SE, Yaniv M (2001). A direct interaction between JNK1 and CrkII is critical for Rac1-induced JNK activation. *The EMBO Journal*.

[40] Nakashima N, Rose DW, Xiao S (1999). The functional role of CrkII in actin cytoskeleton organization and mitogenesis. *Journal of Biological Chemistry*.

[41] Antoku S, Mayer BJ (2009). Distinct roles for Crk adaptor isoforms in actin reorganization induced by extracellular signals. *Journal of Cell Science*.

[42] Han SJ, Lee JH, Hong SH (2002). AP180 binds to the C-terminal SH2 domain of phospholipase C-*γ*1 and inhibits its enzymatic activity. *Biochemical and Biophysical Research Communications*.

[43] Williams CL (2003). The polybasic region of Ras and Rho family small GTPases: a regulator of protein interactions and membrane association and a site of nuclear localization signal sequences. *Cellular Signalling*.

[44] Abassi YA, Vuori K (2002). Tyrosine 221 in Crk regulates adhesion-dependent membrane localization of Crk and Rac and activation of Rac signaling. *EMBO Journal*.

[45] Vallés AM, Beuvin M, Boyer B (2004). Activation of Rac1 by paxillin-Crk-DOCK180 signaling complex is antagonized by Rap1 in migrating NBT-II cells. *Journal of Biological Chemistry*.

[46] Coso OA, Chiarielio M, Yu J-C (1995). The small GTP-binding proteins rac1 and Cdc42 regulate the activity of the JNK/SAPK signaling pathway. *Cell*.

[47] Heller H, Gredinger E, Bengal E (2001). Rac1 inhibits myogenic differentiation by preventing the complete withdrawal of myoblasts from the cell cycle. *Journal of Biological Chemistry*.

[48] Westwick JK, Lambert QT, Clark GJ (1997). Rac regulation of transformation, gene expression, and actin organization by multiple, PAK-independent pathways. *Molecular and Cellular Biology*.

[49] Yang C, Klein EA, Assoian RK, Kazanietz MG (2008). Heregulin *β*1 promotes breast cancer cell proliferation through Rac/ERK-dependent induction of cyclin D1 and p21Cip1. *Biochemical Journal*.

[50] Polyak K, Lee M-H, Erdjument-Bromage H (1994). Cloning of p27(Kip1), a cyclin-dependent kinase inhibitor and a potential mediator of extracellular antimitogenic signals. *Cell*.

[51] Toyoshima H, Hunter T (1994). p27, A novel inhibitor of G1 cyclin-Cdk protein kinase activity, is related to p21. *Cell*.

[52] Bao W, Thullberg M, Zhang H, Onischenko A, Strömblad S (2002). Cell attachment to the extracellular matrix induces proteasomal degradation of p21CIP1 via Cdc42/Rac1 signaling. *Molecular and Cellular Biology*.

[53] Nobes CD, Hawkins P, Stephens L, Hall A (1995). Activation of the small GTP-binding proteins rho and rac by growth factor receptors. *Journal of Cell Science*.

[54] Fine B, Hodakoski C, Koujak S (2009). Activation of the PI3K pathway in cancer through inhibition of PTEN by exchange factor P-REX2a. *Science*.

[55] Han J, Luby-Phelps K, Das B (1998). Role of substrates and products of PI 3-kinase in regulating activation of Rac-related guanosine triphosphatases by Vav. *Science*.

[56] Nimnual AS, Yatsula BA, Bar-Sagi D (1998). Coupling of Ras and Rac guanosine triphosphatases through the Ras exchanger Sos. *Science*.

[57] Okada S, Pessin JE (1996). Interactions between Src homology (SH) 2/SH3 adapter proteins and the guanylnucleotide exchange factor SOS are differentially regulated by insulin and epidermal growth factor. *Journal of Biological Chemistry*.

[58] Weintraub H, Davis R, Tapscott S (1991). The myoD gene family: nodal point during specification of the muscle cell lineage. *Science*.

[59] Meriane M, Roux P, Primig M, Fort P, Gauthier-Rouviere C (2000). Critical activities of Rac1 and Cdc42Hs in skeletal myogenesis: antagonistic effects of JNK and p38 pathways. *Molecular Biology of the Cell*.

